# Antibody and T-Cell Responses 6 Months After Coronavirus Disease 2019 Messenger RNA-1273 Vaccination in Patients With Chronic Kidney Disease, on Dialysis, or Living With a Kidney Transplant

**DOI:** 10.1093/cid/ciac557

**Published:** 2022-08-09

**Authors:** Jan-Stephan F Sanders, A Lianne Messchendorp, Rory D de Vries, Carla C Baan, Debbie van Baarle, Rob van Binnendijk, Dimitri A Diavatopoulos, Daryl Geers, Katharina S Schmitz, Corine H GeurtsvanKessel, Gerco den Hartog, Marcia M L Kho, Marion P G Koopmans, Renate G van der Molen, Ester B M Remmerswaal, Nynke Rots, Ron T Gansevoort, Frederike J Bemelman, Luuk B Hilbrands, Marlies E J Reinders, the VACcination Immune Response Study (RECOVAC) Collaborators, Alferso C Abrahams, Marije C Baas, Pim Bouwmans, Marc A G J ten Dam, Lennert Gommers, Sophie C Frölke, Dorien Standaar, Marieke van der Heiden, Celine Imhof, Yvonne M R Adema, Reshwan K Malahe, Marieken J Boer-Verschragen, Wouter B Mattheussens, Ria Philipsen, Djenolan van Mourik, Susanne Bogers, Laura L A van Dijk, Marc H Hemmelder, Aiko P J de Vries

**Affiliations:** Department of Internal Medicine, Division of Nephrology, University of Groningen, University Medical Center Groningen, Groningen, The Netherlands; Department of Internal Medicine, Division of Nephrology, University of Groningen, University Medical Center Groningen, Groningen, The Netherlands; Department of Viroscience, Erasmus Medical Center, Rotterdam, The Netherlands; Department of Internal Medicine, Nephrology and Transplantation, Erasmus MC Transplant Institute, Erasmus Medical Center, Rotterdam, The Netherlands; Department of Medical Microbiology and Infection Prevention, University Medical Center Groningen, Groningen, The Netherlands; Center for Infectious Disease Control, National Institute for Public Health and the Environment, Bilthoven, The Netherlands; Center for Infectious Disease Control, National Institute for Public Health and the Environment, Bilthoven, The Netherlands; Radboud Center for Infectious Diseases, Radboud University Medical Center Nijmegen, Nijmegen, The Netherlands; Department of Laboratory Medicine, Laboratory of Medical Immunology, Radboud Institute for Molecular Life Sciences, Radboud University Medical Center Nijmegen, Nijmegen, The Netherlands; Department of Viroscience, Erasmus Medical Center, Rotterdam, The Netherlands; Department of Viroscience, Erasmus Medical Center, Rotterdam, The Netherlands; Department of Viroscience, Erasmus Medical Center, Rotterdam, The Netherlands; Center for Infectious Disease Control, National Institute for Public Health and the Environment, Bilthoven, The Netherlands; Department of Internal Medicine, Nephrology and Transplantation, Erasmus MC Transplant Institute, Erasmus Medical Center, Rotterdam, The Netherlands; Department of Viroscience, Erasmus Medical Center, Rotterdam, The Netherlands; Department of Laboratory Medicine, Laboratory of Medical Immunology, Radboud Institute for Molecular Life Sciences, Radboud University Medical Center Nijmegen, Nijmegen, The Netherlands; Department of Experimental Immunology, Amsterdam Infection and Immunity Institute, Amsterdam University Medical Center, University of Amsterdam, Amsterdam, The Netherlands; Center for Infectious Disease Control, National Institute for Public Health and the Environment, Bilthoven, The Netherlands; Department of Internal Medicine, Division of Nephrology, University of Groningen, University Medical Center Groningen, Groningen, The Netherlands; Renal Transplant Unit, Amsterdam UMC, University of Amsterdam, Amsterdam, The Netherlands; Department of Nephrology, Radboud University Medical Center, Radboud Institute for Health Sciences, Nijmegen, The Netherlands; Department of Internal Medicine, Nephrology and Transplantation, Erasmus MC Transplant Institute, Erasmus Medical Center, Rotterdam, The Netherlands

**Keywords:** COVID-19, mRNA-1273 vaccine, chronic kidney disease, kidney transplantation, dialysis

## Abstract

**Background:**

The immune response to COVID-19 vaccination is inferior in kidney transplant recipients (KTRs) and to a lesser extent in patients on dialysis or with chronic kidney disease (CKD). We assessed the immune response 6 months after mRNA-1273 vaccination in kidney patients and compared this to controls.

**Methods:**

A total of 152 participants with CKD stages G4/5 (eGFR <30 mL/min/1.73 m^2^), 145 participants on dialysis, 267 KTRs, and 181 controls were included. SARS-CoV-2 Spike S1 specific IgG antibodies were measured using fluorescent bead-based multiplex-immunoassay, neutralizing antibodies to ancestral, Delta, and Omicron (BA.1) variants by plaque reduction, and T-cell responses by interferon-*γ* release assay.

**Results:**

At 6 months after vaccination, S1-specific antibodies were detected in 100% of controls, 98.7% of CKD G4/5 patients, 95.1% of dialysis patients, and 56.6% of KTRs. These figures were comparable to the response rates at 28 days, but antibody levels waned significantly. Neutralization of the ancestral and Delta variants was detected in most participants, whereas neutralization of Omicron was mostly absent. S-specific T-cell responses were detected at 6 months in 75.0% of controls, 69.4% of CKD G4/5 patients, 52.6% of dialysis patients, and 12.9% of KTRs. T-cell responses at 6 months were significantly lower than responses at 28 days.

**Conclusions:**

Although seropositivity rates at 6 months were comparable to rates at 28 days after vaccination, significantly decreased antibody levels and T-cell responses were observed. The combination of low antibody levels, reduced T-cell responses, and absent neutralization of the newly emerging variants indicates the need for additional boosts or alternative vaccination strategies in KTRs.

**Clinical Trials Registration:**

NCT04741386.

## Teaser

Antibodies and T cells became undetectable in substantial proportions of dialysis and kidney transplant patients at 6 months after coronavirus disease 2019 messenger RNA-1273 vaccination and were also lower in chronic kidney disease patients than in controls. The slopes of these decreases in vaccine-induced immunity were similar among patient groups and controls. Neutralizing antibodies to the Omicron variant were barely detectable.

COVID-19–associated mortality risk is 3- to 4-fold higher in patients with severely impaired kidney function, patients on dialysis, and kidney transplant recipients (KTRs) compared with the general population [1]. Therefore, sustained effectiveness of COVID-19 vaccination in the face of novel emerging variants is of great importance for these patients.

We recently performed a clinical trial with approximately 800 participants to assess the immunogenicity, tolerability, and safety of the mRNA-1273 COVID-19 vaccine in kidney patients [2]. In particular, KTRs showed a combination of low antibody and nondetectable T-cell responses 28 days following the second vaccination. Notably, almost all dialysis patients and patients with chronic kidney disease (CKD G4/5) showed seroconversion, but antibody levels were significantly lower compared with controls [3]. This is in accordance with other smaller studies that described lower seroconversion rates in patients on dialysis and in transplant recipients 28 days after 2 doses of mRNA vaccines [4, 5].

mRNA vaccines have recently been shown to induce durable immunological memory, with protection against newly emerging SARS-CoV-2 variants in healthy individuals [6, 7]. However, especially among older individuals and patients on immunosuppression, antibody levels rapidly wane over a 6-month period [8]. These data underline the importance of long-term follow-up of high-risk patients with kidney disease to assess the need for additional boosts or alternative vaccination strategies.

We studied the concentration of spike S1 binding antibodies; the level of neutralizing antibodies to the ancestral, Delta, and Omicron (BA.1) variants; and T-cell responses at 6 months after mRNA-1273 COVID-19 vaccination in patients with severely impaired kidney function, patients on dialysis, KTRs, and controls without known kidney disease.

## METHODS

The design of the Dutch Renal patients COVID-19 VACcination (RECOVAC) Immune Response study was published previously [2]. Ethical approval was obtained from the Dutch Central Committee on Research Involving Human Subjects and the local ethics committees of the participating centers.

### Study Participants and COVID-19 Vaccination

Four cohorts were included in the study. Cohort A (controls) consisted of participants without kidney disease (eGFR >45 mL/min/1.73 m^2^), cohort B of patients with severely impaired kidney function (eGFR <30 mL/min/1.73 m^2^ or CKD stages G4/5), cohort C of patients on hemodialysis or peritoneal dialysis, and cohort D of KTRs. The control cohort included partners, siblings, or household members of participants in cohorts B, C, and D. All participants received 2 mRNA-1273 COVID-19 vaccinations (Moderna Biotech Spain, S.L.) with an interval of 28 days. Blood samples were collected at baseline, prior to the second vaccination, and 28 days and 6 months after the second vaccination. Patients who experienced COVID-19 before or during the study were excluded.

### SARS-CoV-2 Spike S1-Specific Immunoglobulin G Antibody Response and Virus Neutralizing Antibodies

SARS-CoV-2 spike S1-specific IgG antibodies were measured in serum samples using a validated fluorescent bead-based multiplex-immunoassay, as previously described, and expressed as international binding antibody units per milliliter (BAU/mL) [9, 10]. Participants were classified as seropositive or seronegative; the cutoff was set at S1-specific IgG antibody concentration ≥10 BAU/mL [10, 11]. Nucleocapsid antibodies were measured at all time points using multiplex immunoassay, as previously described, and classified as positive or negative [12].

Plaque reduction neutralization tests against the ancestral SARS-CoV-2 and the Delta and Omicron variants were performed as described previously [7, 11]. For feasibility, it was a priori decided to measure neutralizing antibodies only in a random sample of 20 patients per group with measurable S1-specific IgG antibodies at 6 months included in 1 of the participating centers (Erasmus MC Rotterdam).

### SARS-CoV-2–Specific T-Cell Response

SARS-CoV-2–specific T-cell responses were measured in all patients participating at the Erasmus MC Rotterdam including 40 controls, 36 CKD G4/5 patients, 38 dialysis patients, and 62 KTRs. Measurement was performed using a commercially available interferon (IFN)-*ɣ* release assay (IGRA) according to the manufacturer's instructions (QuantiFERON, QIAGEN) [13]. Results from the Antigen-2 (peptides covering the entire S protein) assay were expressed in IU/mL after subtraction of the negative control values as interpolated from a standard calibration curve.

### Antibody Decay

Previous studies have shown that decay of SARS-CoV-2–specific antibodies most likely follows an exponential pattern over time [14]. We therefore calculated decay in antibodies over time using an exponential formula in order to estimate antibody half-life and time to reach certain antibody levels as follows: *y* = *a* × *b^X^*, where *y* is the S1 IgG antibody level at 6 months, *a* is the S1 IgG antibody level at 28 days, *b* is the slope, and *X* is time. The slope *b* was calculated as: log10(*b*) = (log10(*y*) − log10(*a*))/(X(*y*) − X(*a*)). Half-life was subsequently calculated as: half-life = log10(.5)/log10(*b*). Time to reach a certain S1 IgG antibody level (*c*) was calculated as: time until *c* = *X*(*a*) + (log10(*c*) − log10(*a*))/log10(*b*).

### Statistical Analyses

Continuous data are presented as mean with standard deviation or as median and interquartile interval in case of nonnormal distribution. Categorical data are presented as percentages. Differences between patient groups and the control group were tested using the independent *t* test, Mann–Whitney *U* test, or Pearson *χ*^2^ test depending on data distribution, with Bonferroni correction for multiple testing. Differences within study cohorts over time were tested using the paired sample *t* test, Wilcoxon signed rank test, or Pearson *χ*^2^ test depending on data distribution. The correlation between the S1 IgG antibody levels measured at 28 days and 6 months after the second vaccination was tested by performing Pearson correlation. All analyses were performed with the statistical software IBM SPSS Statistics version 23.0 (SPSS Inc, Chicago, IL). Figures were created with the software GraphPad Prism version 5.00 (GraphPad Software, San Diego, CA). A 2-sided *P* value <.05 was adopted to denote statistical significance and corrected in case of multiple testing using Bonferroni correction unless stated otherwise.

## RESULTS

### Baseline Characteristics

**Figure 1.**
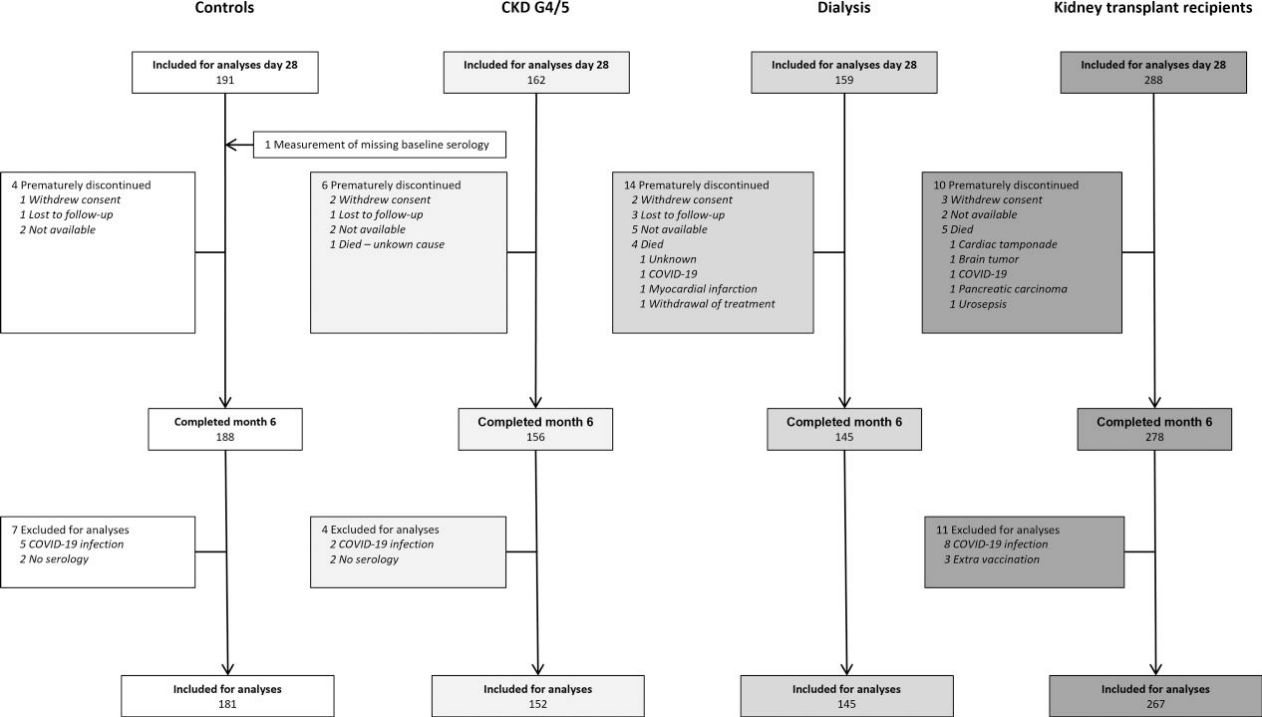
Participant enrollment and outcomes 6 months after second vaccination. Abbreviations: CKD G4/5, chronic kidney disease stages 4/5; COVID-19, coronavirus disease 2019.

A flowchart of study enrollment is depicted in Figure 1. In total, 181 controls, 152 patients with CKD G4/5, 145 dialysis patients, and 267 KTRs were included for the analysis of binding antibody levels 6 months after vaccination. Baseline characteristics of these participants are shown in Table 1.

**Table 1.** Baseline Characteristics per Study Group

Characteristic	Control (n = 181)	Chronic Kidney Disease Stages 4/5 (n = 152)	Dialysis (n = 145)	Kidney Transplant Recipient (n = 267)
Female, n (%)	107 (59.1)	53 (34.9)	48 (33.1)	123 (46.1)
White, n (%)	167 (92.3)	135 (88.8)	121 (83.4)	243 (91.0)
Age, years	58.4 ± 12.9	60.6 ± 13.4	60.0 ± 13.8	55.9 ± 14.1
Body mass index, kg/m^2^	27.5 ± 5.3	27.8 ± 5.2	26.7 ± 5.7	27.0 ± 4.6
Systolic blood pressure, mm Hg	146.3 ± 22.7	151.1 ± 24.1	139.2 ± 25.8	146.4 ± 21.3
Diastolic blood pressure, mm Hg	84.8 ± 11.6	84.1 ± 11.9	78.2 ± 16.5	84.5 ± 10.9
Current smoking, n (%)	31 (17.1)	23 (15.1)	33 (22.8)	28 (10.5)
Current alcohol consumption, n (%)	108 (59.7)	60 (39.5)	32 (22.1)	105 (39.3)
Number of comorbidities	0 (0−1)	1 (1−2)	1 (1−2)	1 (1−2)
Comorbidity, n (%)				
ȃHypertension	49 (27.1)	126 (82.9)	96 (66.2)	219 (82.0)
ȃDiabetes mellitus	17 (9.4)	40 (26.3)	35 (24.1)	56 (21.0)
ȃHistory of coronary artery disease	9 (5.0)	33 (21.7)	33 (22.8)	35 (13.1)
ȃHeart failure	2 (1.1)	12 (7.9)	10 (6.9)	13 (4.8)
ȃChronic lung disease	14 (7.7)	16 (10.5)	14 (9.7)	12 (4.5)
ȃHistory of malignancy^a^	9 (5.0)	20 (13.2)	34 (23.4)	40 (15.0)
ȃAutoimmune disease	4 (2.2)	3 (2.0)	5 (3.4)	14 (5.2)
Lymphocytes, 10^9^/L	2.0 (1.6−2.5)	1.6 (1.2−2.0)	1.2 (0.9−1.6)	1.3 (0.9−1.9)
Estimated glomerular filtration rate, mL/min/1.73 m^2^	82.3 ± 18.5	17.7 ± 6.1	...	49.5 ± 18.9
Primary renal diagnosis, n (%)				
ȃPrimary glomerulonephritis	...	18 (11.8)	14 (9.7)	53 (19.9)
ȃPyelonephritis	...	1 (0.7)	1 (0.7)	54 (1.5)
ȃInterstitial nephritis	...	7 (4.6)	4 (2.8)	9 (3.4)
ȃFamilial/hereditary renal diseases	...	25 (16.4)	19 (13.1)	51 (19.1)
ȃCongenital diseases	...	6 (3.9)	5 (3.4)	18 (6.7)
ȃVascular diseases	...	31 (20.4)	27 (18.6)	26 (9.7)
ȃSecondary glomerular/systemic disease	...	4 (2.6)	7 (4.8)	12 (4.5)
ȃDiabetic kidney disease	...	9 (5.9)	21 (14.5)	10 (3.7)
ȃOther	...	29 (19.1)	24 (16.6)	39 (14.6)
ȃUnknown	...	22 (14.4)	23 (15.9)	45 (16.8)
Dialysis characteristics, n (%)				
ȃHemodialysis	...	...	110 (75.9)	...
ȃPeritoneal dialysis	...	...	35 (24.1)	...
ȃTime on dialysis, months	...	...	30.0 (14.0−69.8)	...
Transplant characteristics				
ȃFirst kidney transplant, n (%)	...	...	...	208 (77.9)
ȃTime after last transplantation, years	...	...	...	6.0 (2.0−13.0)
Last transplant				
ȃȃLiving, n (%)	...	...	...	183 (68.5)
ȃȃPreemptive, n (%)	...	...	...	98 (36.7)
Number of immunosuppressive agents	...	...	...	2 (2−3)
Immunosuppressive treatment at baseline, n (%)				
ȃSteroids	...	...	...	203 (76.0)
ȃAzathioprine	...	...	...	32 (12.0)
ȃMycophenolate mofetil	...	...	...	183 (68.5)
ȃCalcineurin inhibitor	...	...	...	221 (82.8)
ȃmTOR inhibitor	...	...	...	17 (6.4)
ȃOther	...	...	...	5 (1.9)
ȃInduction with rituximab last year, n (%)	...	...	...	2 (0.7)
Received kidney transplant after baseline, n (%)	...	13 (8.6)	13 (9.0)	1 (0.4)
Start dialysis after baseline, n (%)	...	9 (5.9)	...	1 (0.4)
Immunosuppressive treatment at month 6, n (%)				
ȃSteroids	...	13 (8.6)	11 (7.6)	196 (73.4)
ȃAzathioprine	...	...	1 (0.7)	31 (11.6)
ȃMycophenolate mofetil	...	11 (7.2)	9 (6.2)	181 (67.8)
ȃCalcineurin inhibitor	...	13 (8.6)	11 (7.6)	214 (80.1)
ȃmTOR inhibitor	...	2 (1.3)	2 (1.4)	17 (6.4)

Variables are presented as mean ± standard deviation or as median (interquartile interval) in case of nonnormal distribution.

Abbreviation: mTOR, mechanistic target of rapamycin.

Including melanomas, excluding all other skin malignancies.

### SARS-CoV-2 Spike S1-Specific IgG Antibody Response at 6 Months

**Figure 2.**
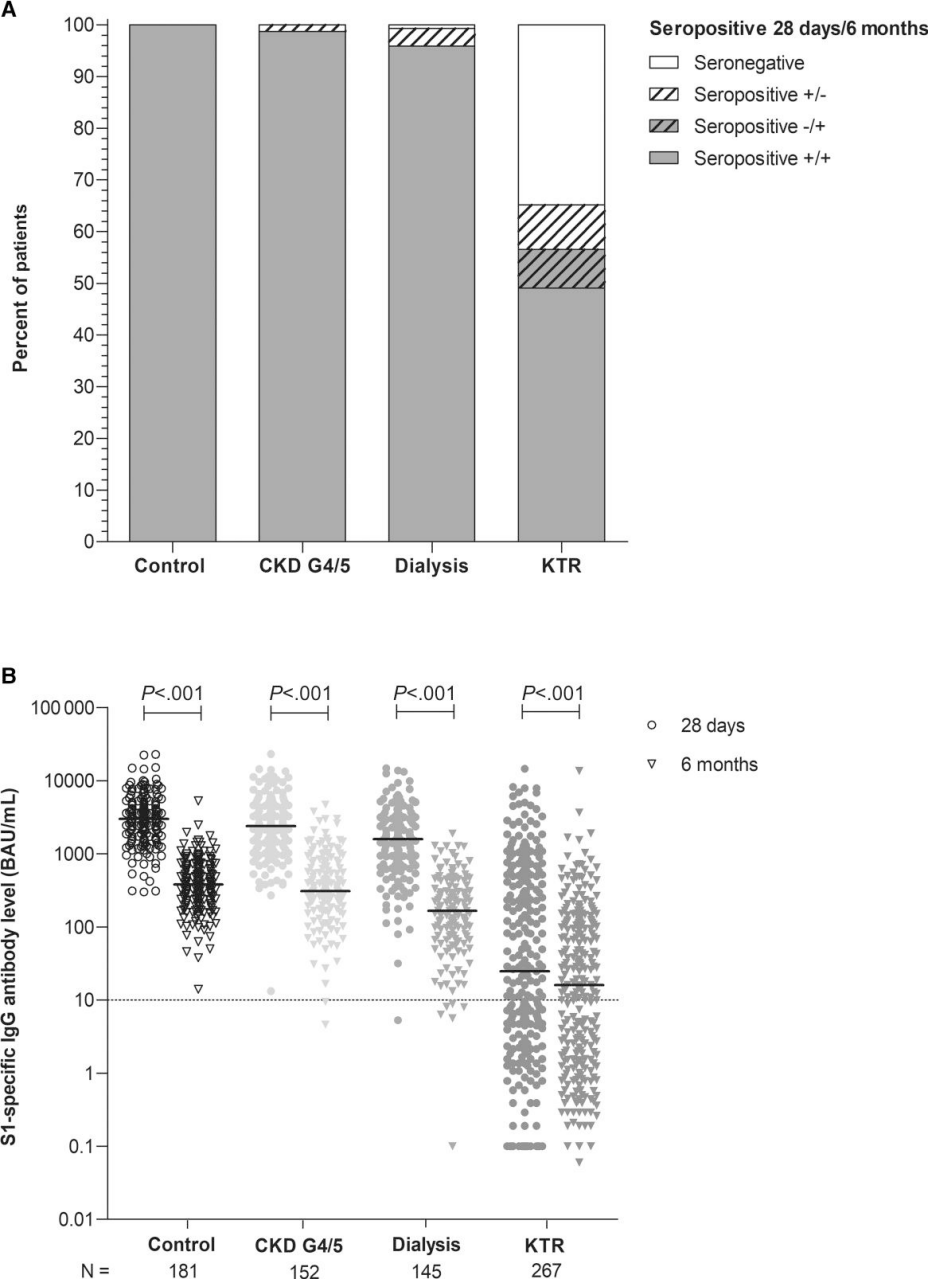
Seroconversion rate *(A)* and S1-specific IgG antibody levels *(B)* at day 28 and month 6 after second coronavirus disease 2019 vaccination per cohort. Depicted are scatter dot plots with a line indicating the median level. *P* values were calculated using the Wilcoxon signed rank test. Abbreviations: BAU/mL, binding antibody units per milliliter; CKD G4/5, chronic kidney disease stages 4/5; IgG, immunoglobulin G; KTR, kidney transplant recipient.

All controls retained seropositivity 6 months after vaccination. The seropositivity rates in patients with CKD G4/5 and patients on dialysis were 98.7% and 95.1%, respectively (compared with 100% and 99.3% at day 28). In KTRs, the seropositivity rate was 57.7% at day 28; of these patients, 14.9% (n = 23) became seronegative at 6 months (*P* < .001). Remarkably, 17.7% (n = 20) of seronegative KTRs at day 28 became seropositive at 6 months (Figure 2*A*). Overall, 56.6% of KTRs were seropositive at month 6.

In all 4 groups, the S1-specific IgG antibody levels declined significantly from day 28 to month 6, but with good correlation between the 2 time points (*R* = 0.88, *P* < .001) (Supplementary Figure 1). In controls, levels decreased 7.7-fold from 3009 to 380 BAU/mL. In CKD G4/5 patients, levels decreased 7.5-fold from 2380 to 309 BAU/mL; in dialysis patients, 9-fold from 1585 to 165 BAU/mL; and in KTRs, 2.3-fold from 25 to 16 BAU/mL (all *P* < .001; Figure 2*B*). At 6 months after vaccination, S1-specific IgG antibody levels in dialysis patients and KTRs were significantly lower when compared with controls (both *P* < .001). While all CKD G4/5 patients, dialysis patients, and controls had a decrease in S1-specific IgG antibody levels, 32 KTRs (18%) had an increase in S1-specific IgG antibody levels. None of these patients reported having contracted COVID-19 or had detectable nucleocapsid antibodies.

### Decay in S1-Specific IgG Antibodies and Predictors of Decay

**Figure 3.**
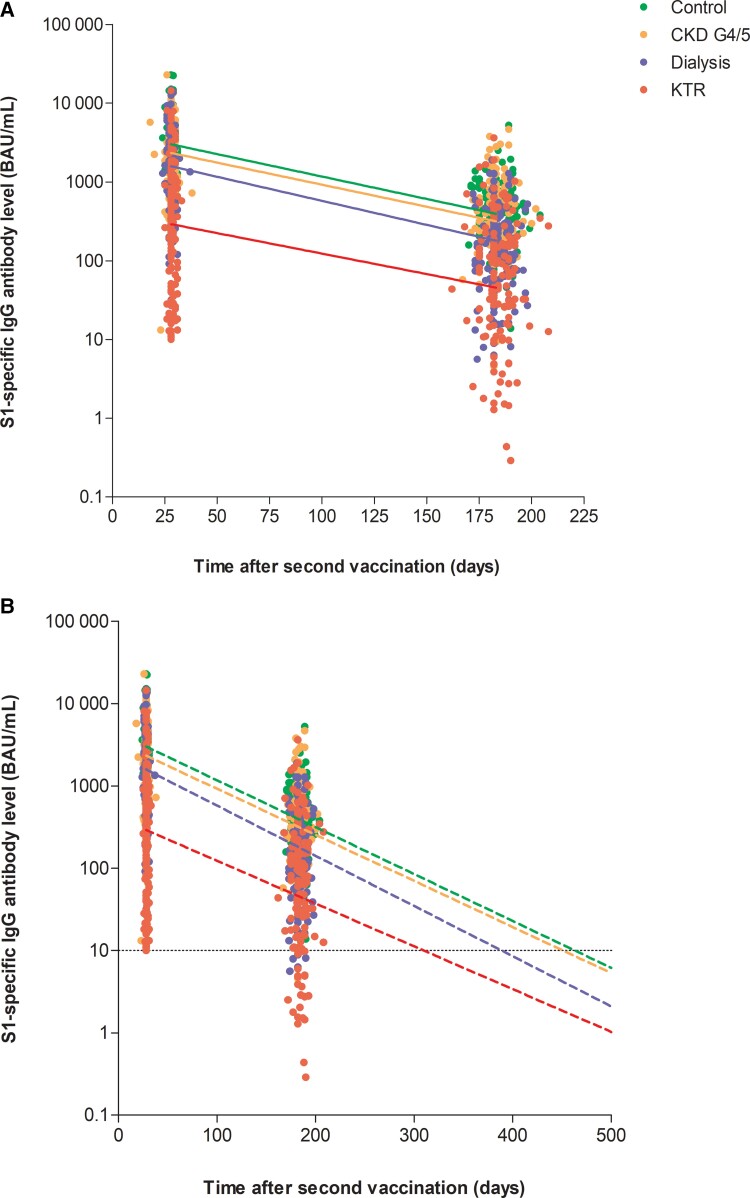
Decay in antibodies according to exponential decay model. *A*, Decay per group in participants with antibody value at day 28 ≥10 BAU/mL. *B*, Estimated time until 10 BAU/mL. Abbreviations: BAU/mL, binding antibody units per milliliter; CKD G4/5, chronic kidney disease stages 4/5; IgG, immunoglobulin G; KTR, kidney transplant recipient.

Since significant waning of S1-specific IgG antibody levels was detected at 6 months after vaccination in all groups, we calculated the antibody half-life assuming an exponential decay. The overall half-life in the entire cohort was 52 days (41–69), and this was comparable between the 4 groups (Table 2, Figure 3). With an exponential decay model, we calculated the time until seronegativity. This time was 451 days after the second vaccination in controls and 442 days in CKD G4/5 patients, whereas it was significantly shorter at 381 days in dialysis patients and 308 days in KTRs (both *P* < .001 compared with the control group). Several characteristics differed significantly between participants in whom S1-specific IgG antibody levels declined faster (half-life below 52 days) or slower (half-life above 52 days) (Supplementary Table 1), but these characteristics were not consistent between the different groups.

**Table 2.** Decay in S1-Specific Immunoglobulin G Antibody Level per Study Group Assuming an Exponential Decay

Variable	Control	Chronic Kidney Disease Stages 4/5	Dialysis	Kidney Transplant Recipient
Half-life, days	52.6 (43.2–65.5)	52.4 (43.4–72.6)	49.6 (40.4–63.5)	52.9 (32.7–81.3)
Seroresponse defined as ≥10 BAU/mL at day 28, n (%)	181 (100)	152 (100)	144 (100)	154 (57.7)
S1 immunoglobulin G level (BAU/mL) in seropositive participants	3009 (1812–4797)	2380 (1267–4569)	1587 (702–3121)^a^	310 (57.1–1041)^a^
Time to 10 BAU/mL (days) in seropositive participants	451 (378–569)	442 (368–610)	381 (313–494)^a^	308 (119–473)^a^

Variables are presented as mean ± standard deviation, as median (interquartile interval) in case of nonnormal distribution, or as number (percentage) in case of categorical data. *P* values were calculated using the Mann–Whitney *U* test with the control group as the reference group. Bonferroni correction was applied for multiple testing.

Abbreviation. BAU/mL, binding antibody units per milliliter.

*P* < .001.

### Neutralizing Antibodies Targeting SARS-CoV-2 Variants

**Figure 4.**
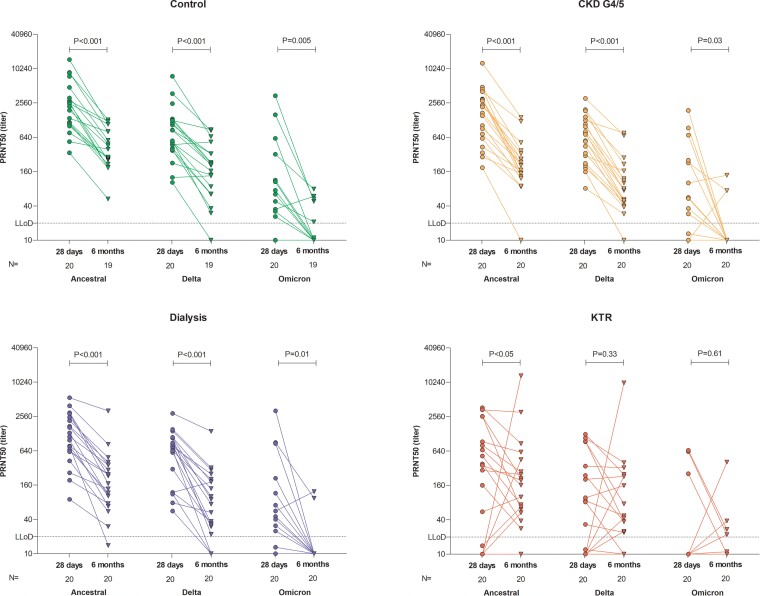
Levels of neutralizing antibodies against the ancestral SARS-CoV-2 (wild type) and the recently emerged Delta and Omicron variants per subgroup and compared with level of neutralizing antibodies at 6 months. The dotted horizontal line indicates the lower limit of detection of neutralization (titer of 20). *P* values were calculated using the Wilcoxon signed rank test. Abbreviations: CKD G4/5, chronic kidney disease stages 4/5; KTR, kidney transplant recipient; LLoD, lower limit of dectection; PRNT50, 50% plaque reduction neutralization test.

In a selection of 20 participants per group with S1-specific IgG antibodies, neutralization against the ancestral SARS-CoV-2 and the Delta and Omicron (BA.1) variants was assessed (Figure 4). Neutralizing antibodies to the ancestral and Delta SARS-CoV-2 strains were detected in all controls, CKD G4/5 patients, and patients on dialysis at 28 days (Figure 4). At 6 months, in several participants in the control, CKD G4/5, and dialysis groups, levels of Delta virus neutralizing antibodies had dropped below the detection levels. Levels of neutralizing antibodies were significantly lower at 6 months compared with 28 days after the second vaccination (Figure 4). In KTRs, not all participants showed neutralizing antibodies against the ancestral and Delta variants. Notably, waning of neutralizing antibodies was not apparent in KTRs at 6 months compared with 28 days post vaccination. Levels of neutralizing antibodies against the ancestral strain and Delta strain correlated with the levels of S1-specific IgG antibodies at 28 days and 6 months after the second vaccination (ancestral/wild type *R* = .88 and *R* = 0.85, respectively, both *P* < .001; Delta *R* = 0.83 and *R* = 0.85, respectively, both *P* < .001; Supplementary Figure 2*A* and 2*B*). Notably, neutralization of the newly emerged Omicron variant was barely detectable in any of the groups, both at 28 days and 6 months after vaccination. Correlation plots of neutralizing antibodies against Omicron and S1-specific IgG antibodies showed that Omicron was only neutralized at high titers of S1-specific IgG, both at 28 days and 6 months after the second vaccination (*R* = 0.51 and *R* = 0.55, both *P* < .001; Supplementary Figure 2*C*).

### SARS-CoV-2–Specific T-Cell Responses at 6 Months

**Figure 5.**
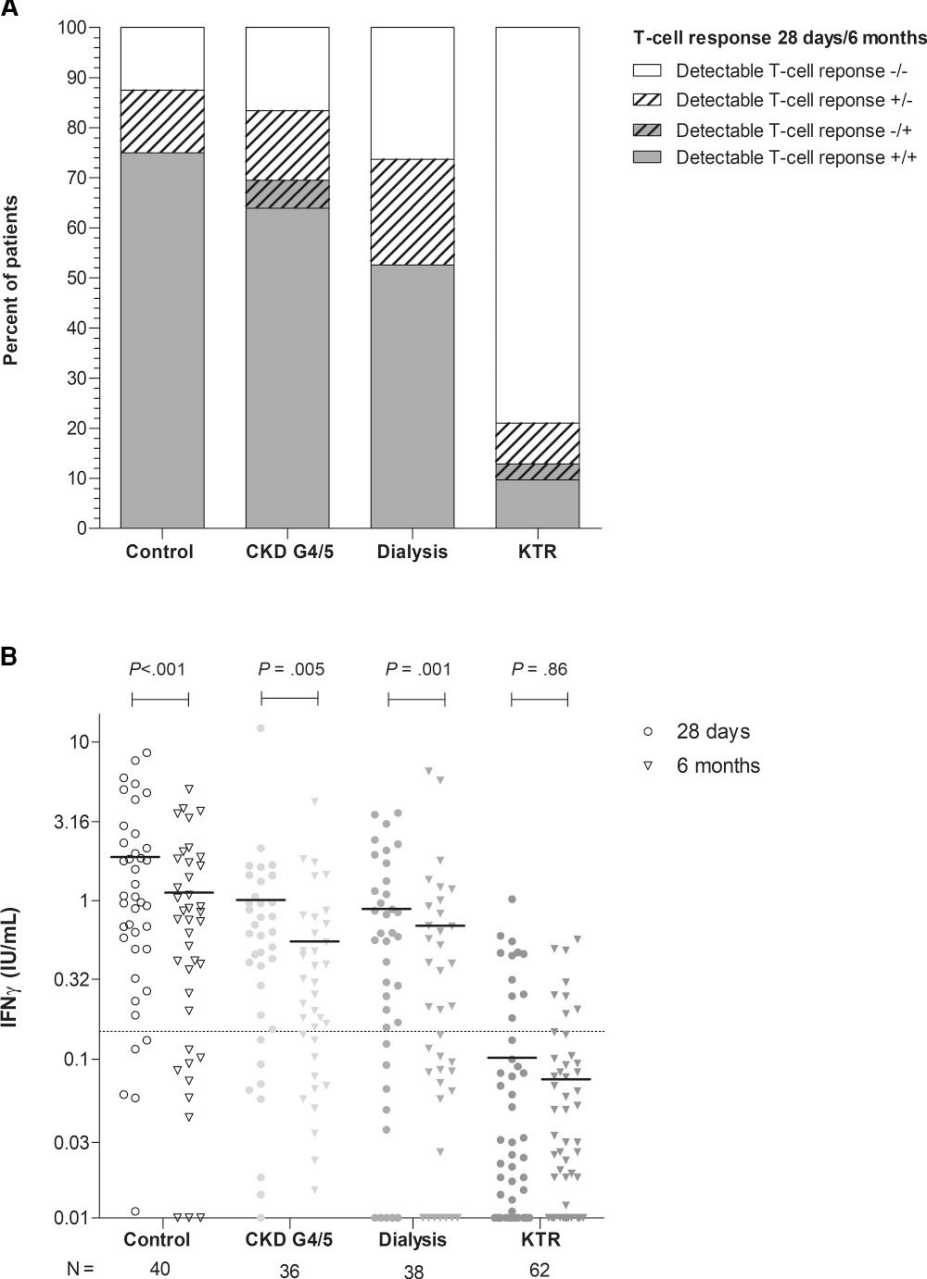
SARS-CoV-2–specific T-cell response in all participants at 1 of the participating centers. *A*, Percentage of T-cell responders per group 6 months after vaccination (defined as Antigen-2 ≥0.15 IU/mL). *B*, Individual IFN-*γ* levels per group, with the horizontal line representing the median value. Dotted horizontal line indicates the threshold of detectable T-cell response (≥0.15 IU/mL). *P* values were calculated using the Wilcoxon signed rank test. Abbreviations: CKD G4/5, chronic kidney disease stages 4/5; IFN-*γ*, interferon gamma; KTR, kidney transplant recipient.

CoV-2–specific T-cell response (defined as IFN-*ɣ* concentration ≥0.15 IU/mL after specific stimulation) was observed in 87.5% and 75.0% of controls, 77.8% and 69.4% of CKD G4/5 patients, and 73.3% and 52.6% of dialysis patients at 28 days and 6 months, respectively (*P* < .001, *P* = .002, and *P* < .001, respectively; Figure 5*A*). A detectable T-cell response was observed in 17.7% of KTRs at day 28; of these patients, 45.5% (n = 5) had a nondetectable T-cell response at 6 months (*P* < .001).

T-cell responses correlated to the levels of S1-specific binding antibodies, both at 28 days and 6 months after the second vaccination (*R* = 0.64 and *R* = 0.59, respectively, both *P* < .001; Supplementary Figure 3). The median IFN-*ɣ* level at 6 months tended to be lower in CKD G4/5 patients (0.28 IU/mL) and were significantly lower in dialysis patients (0.21 IU/mL) compared with controls (0.76 IU/mL; *P* = .06 and *P* = .04, respectively). Median IFN-*ɣ* levels were also significantly lower in KTRs at 0.02 IU/mL when compared with controls (*P* < .001). Nevertheless, in KTRs, median IFN-*ɣ* levels at 6 months were not significantly lower vs at 28 days, opposed to an observed significant decline in controls, CKD G4/5 patients, and dialysis patients (Figure 5*B*).

Of 62 KTRs, 3 (4.8%) showed an increase in IGRA response, defined as >0.15 IU/mL at both time points, with doubling between day 28 and month 6. An increase in SARS-CoV-2–specific T-cell response between day 28 and month 6 was observed in 2 controls (5.0%), 4 CKD G4/5 patients (11.1%), and no dialysis patients. There was no relation between a rise in S1 antibody titers and an increase in T-cell response in the KTRs.

### Safety

Overall, 25 safety events were reported between day 28 and month 6. Significantly more safety events occurred in dialysis patients and KTRs compared with controls (6.2% and 4.5% vs 0%, *P* = .003 and *P* = .01, respectively). None were classified as related to COVID-19 vaccination (Table 3). In total, 10 patients died due to variable causes; none of these cases were classified as related to COVID-19 vaccination. Finally, in the KTR group, 1 participant experienced allograft rejection, which was not related to COVID-19 vaccination, and recovered after treatment with methylprednisolone and subsequently thymoglobulin.

**Table 3.** List of Serious Adverse Events Between 28 Days and 6 Months After Second Vaccination

Serious Adverse Events	Control (N = 181)	Chronic Kidney Disease Stages 4/5 (N = 152)	Dialysis (N = 145)	Kidney Transplant Recipient (N = 267)
Any serious adverse event, n (%)	0	4 (2.6)	9 (6.2)	12 (4.5)
Related to vaccination, n (%)	...	0	0	0
Not related to vaccination, n (%)				
ȃUrinary tract infection	...	2 (1.3)	1 (0.7)	7 (2.6)
ȃMalaise	...	2 (1.3)	...	1 (0.4)
ȃColitis	...	...	1 (0.7)	...
ȃFever	...	...	2 (1.4)	2 (0.7)
ȃFluid overload	...	...	1 (0.7)	...
ȃSyncope	...	...	1 (0.7)	...
ȃCholedocholithiasis	...	...	1 (0.7)	...
ȃInfected hematoma	...	1 (6.6)	1 (0.7)	...
ȃVaricella zoster infection	...	...	...	1 (0.4)
ȃAcute renal insufficiency	...	...	...	1 (0.4)
ȃFemoropopliteal artery bypass thrombosis	...	...	...	1 (0.4)
ȃAbdominal pain	...	...	1 (0.7)	...
ȃRectal blood loss	...	...	1 (0.7)	...
ȃAmputation	...	...	1 (0.7)	...
ȃHypocalcemia	...	...	...	1 (0.4)
ȃAcute renal rejection	...	...	...	1 (0.4)
ȃ Post-transplantation lymphoproliferative disorder	...	...	1^a^ (0.7)	...
ȃ Cytomegalovirus infection	...	...	1 (0.7)	...

Variables are given as number and percentage. *P* values were calculated using the *χ*^2^ test.

Participant received a kidney transplant after baseline visit.

## DISCUSSION

In this study, we demonstrate waning of binding antibodies, neutralizing antibodies, and T-cell responses in different groups of kidney patients at 6 months after vaccination with the mRNA-1273 COVID-19 vaccine. The slopes of these decreases in vaccine-induced immunity were similar among patient groups and controls. Consequently, SARS-CoV-2–specific antibodies and T cells became undetectable in a substantial proportion of dialysis patients, especially KTRs, when compared with controls. At 6 months after vaccination, neutralizing antibodies to the circulating Omicron variant were barely detectable in any of the groups.

Until now, data on durability of the response to vaccination were not available from adequately powered and controlled vaccination studies in kidney patients, although stronger waning of antibodies in dialysis patients was previously observed [15]. Additionally, a study in 312 solid organ transplant recipients showed that seropositivity rates after mRNA vaccination remained relatively stable until 6 months [16]. Interestingly, similar to our observations, that study also detected an increase in seropositivity in 43 solid organ transplant recipients (14.7%), but the authors could not exclude asymptomatic infections as a cause of this increase. This phenomenon of increasing antibody concentrations was also observed by Hall et al in a small subgroup of transplant recipients who received placebo vaccination but nevertheless had an increase in anti-Receptor Binding Domain antibody levels [17]. We detected an increase in antibody concentration in 18% of the KTRs between 28 days and 6 months after vaccination, while these participants had not reported SARS-CoV-2 infection and we did not detect nucleocapsid-specific antibodies. This late increase in antibody levels could be explained by ongoing delayed mRNA vaccine–induced B-cell stimulation and/or delayed plasma cell differentiation in KTRs [18].

Neutralizing antibodies are regarded as an important correlate of protection against developing severe COVID-19 [19, 20]. We show that the majority of kidney patients with measurable binding antibodies can still neutralize both the ancestral SARS-CoV-2 variant, as well as the Delta variant. Neutralizing antibodies were significantly lower in KTRs, although waning was less pronounced in this group. In accordance with current literature, cross-neutralization of the emerging Omicron variant (BA.1) was strongly reduced, and almost none of the sera obtained 6 months after vaccination neutralized this variant [7, 21–23].

The absence of cross-neutralization explains that reinfections and breakthrough infections with the Omicron variant are now frequently seen. The Omicron (BA.1) variant is the first variant that formed a novel antigenic cluster [24], explaining the reduced vaccine efficacy against this variant. Fortunately, a lower risk of severe disease after infection with this variant was described [25]. This is potentially due to inherent differences in viral properties between the Omicron and previously circulating variants. The absence of cross-neutralization of Omicron suggests that immunological mechanisms other than virus neutralization are involved in cross-protection against severe disease. These might include effector functions mediated by nonneutralizing antibodies and virus-specific T cells. We therefore speculate that the combination of low binding antibody levels and reduced T-cell responses, in conjunction with lack of neutralization of the Omicron variant, as observed in KTRs, could predispose to more severe disease upon infection with the Omicron variant.

Thus far, limited data on virus-specific T-cell responses in high-risk groups of kidney patients have been reported, especially during standardized follow-up. In healthy individuals, cell-mediated immune responses are detectable up to at least 8 months after vaccination [6, 7]. We observed a trend toward waning T-cell responses in this study. However, compared with the antibody concentrations, T-cell responses were relatively stable. T-cell responses were undetectable in the peripheral blood obtained from the majority of KTRs 6 months after vaccination. However, the fact that we could not measure SARS-CoV-2–specific T cells in the circulation does not exclude that functional T cells could have been present in the lymphoid organs to play a role in B-cell activation and antibody production. An in-depth analysis of virus-specific T cells is required to better understand the differences in phenotype and functionality of T cells between groups.

We report the immunogenicity of mRNA-1273 vaccination 6 months after completion of the 2-shot regimen, but a third vaccination had already been implemented in clinical practice in high-risk groups. Our data show that 6 months after the second vaccination, especially in KTRs, the levels of neutralizing antibodies against the ancestral and emerging variants as well as T-cell responses are low or not detectable. This underlines the importance of the third vaccination as a standard part of a complete COVID-19 vaccination schedule for KTRs. Moreover, when an increased COVID-19 risk exists, this vulnerable patient group should be protected by masks and social distancing as advised by the Centers for Disease Control and Prevention [26]. Finally, AZD7442 (Evusheld) could be considered as preexposure prophylaxis in severely immunocompromised patients. Additionally, our data show a similar half-life of antibodies in all groups. Although seroconversion rates were high in CKD G4/5 and dialysis patients, antibody levels in these patient groups were lower than in controls. This suggests that the interval between the second and third vaccination should be shorter in these patients than in the general population.

The main strength of our study is the prospective design with the inclusion of different kidney patients as well as a control cohort. The study assessed both (functional) antibody responses as well as T-cell responses at predefined fixed time points using standardized assays. Study limitations include that all patients received the mRNA-1273 (Moderna) COVID-19 vaccine, which precludes conclusions about the response to other vaccines, and that patients using immunosuppressive therapy were excluded at baseline from the CKD G4/5 as well as dialysis cohorts, which may have skewed the seroconversion rate and waning of antibodies in these patients. On the other hand, this enabled specific evaluation of the role of impaired kidney function and kidney function replacement treatment.

In conclusion, although seropositivity rates at 6 months after vaccination were comparable to response rates 28 days after vaccination, significantly decreased antibody levels and T-cell responses were observed in all groups. In particular, KTRs displayed a combination of low antibody levels, few detectable T-cell responses, and lack of neutralization of circulating variants 6 months after vaccination. Alternative strategies to improve immunogenicity of COVID-19 vaccines, including additional boosts with (variant-adapted) COVID-19 vaccines, should be considered to reduce the risk of severe disease in these vulnerable patients.

## Supplementary Data

Supplementary materials are available at *Clinical Infectious Diseases* online. Consisting of data provided by the authors to benefit the reader, the posted materials are not copyedited and are the sole responsibility of the authors, so questions or comments should be addressed to the corresponding author.

## Supplementary Material

ciac557_Supplementary_Data
