# Supplementary material for: Antibody and T-Cell Responses 6 Months After Coronavirus Disease 2019 Messenger RNA-1273 Vaccination in Patients With Chronic Kidney Disease, on Dialysis, or Living With a Kidney Transplant
Source: Clin Infect Dis. 2022 Aug 9;76(3):e188–99. doi: 10.1093/cid/ciac557 (PMC9278186; doi:10.1093/cid/ciac557)
Supplement: ciac557_Supplementary_Data [file ciac557_supplementary_data.docx]

**- Supplementary Data -**

**Antibody and T-cell responses 6 months after COVID-19 mRNA-1273 vaccination in patients with chronic kidney disease, on dialysis, or living with a kidney transplant**

^1^Jan-Stephan F Sanders, M.D., Ph.D., Ph.D., ^1^A Lianne Messchendorp, M.D., Ph.D.^#^, ^2^Rory D de Vries, Ph.D.^#^, ^3^Carla C Baan, Ph.D., ^4,5^Debbie van Baarle, Ph.D., ^5^Rob van Binnendijk, Ph.D., ^6,7^Dimitri A Diavatopoulos, Ph.D., ^2^Daryl Geers, M.Sc., ^2^Katharine S. Schmitz, M.Sc., ^2^Corine H. GeurtsvanKessel, M.D., Ph.D., ^5^Gerco den Hartog Ph.D*.,* ^3^Marcia ML Kho, M.D., ^2^Marion PG Koopmans, ^7^Renate G. van der Molen, Ph.D., ^8^Ester BM Remmerswaal, Ph.D, ^5^Nynke Rots, Ph.D., ^1^Ron T Gansevoort, M.D., Ph.D., ^9^Frederike J Bemelman, M.D.^#^, ^10^Luuk B Hilbrands, M.D., Ph.D.^#^, ^3^Marlies EJ Reinders, M.D., Ph.D.^#^, RECOVAC Collaborators*

^1^ Department of Internal Medicine, Division of Nephrology, University of Groningen, University Medical Center Groningen, Groningen, the Netherlands

^2^ Department Viroscience, Erasmus Medical Center, Rotterdam, the Netherlands

^3^ Department of Internal Medicine, Nephrology and Transplantation, Erasmus MC Transplant Institute, Erasmus Medical Center, Rotterdam, The Netherlands

^4^ Department of Medical Microbiology and Infection Prevention, University Medical Center Groningen, Groningen, The Netherlands

^5^ Center for Infectious Disease Control, National Institute for Public Health and the Environment, Bilthoven, The Netherlands

^6^ Radboud Center for Infectious Diseases, Radboud University Medical Center Nijmegen, Nijmegen, The Netherlands

^7^ Radboud Institute for Molecular Life Sciences, Department of Laboratory Medicine, Laboratory of Medical Immunology, Radboud University Medical Center Nijmegen, Nijmegen, The Netherlands

^8^ Department of Experimental Immunology, Amsterdam Infection and Immunity Institute, Amsterdam UMC, University of Amsterdam, Amsterdam, The Netherlands

^9^ Renal Transplant Unit, Amsterdam UMC, University of Amsterdam, Amsterdam, The Netherlands

^10^ Department of Nephrology, Radboud University Medical Center, Radboud Institute for Health Sciences, Nijmegen, The Netherlands

* A list of RECOVAC Collaborators is added in the Acknowledgements

^#^ contributed equally

**Table S1.** Differences in subject characteristics between patients with CKD4/5, dialysis and KTR with calculated half-life of S1-specific IgG antibody level ≥52 days and <52 days after the second vaccination with S1-specific IgG antibody level of ≥10 BAU/mL 28 days after second vaccination.

|  | **CKD G4/5** | |  | **Dialysis** | |  | **KTR** | |
| --- | --- | --- | --- | --- | --- | --- | --- | --- |
| **Half-life of antibodies (days)** | ≥52  (N=78) | <52  (N=74) |  | ≥52  (N=67) | <52  (N=77) |  | ≥52  (N=78) | <52  (N=76) |
| Female, n (%) | **31 (39.7)** | **22 (29.7)^*^** |  | **28 (41.8)** | **20 (26.0)^**^** |  | 36 (46.2) | 30 (39.5) |
| Caucasian, n (%) | 70 (89.7) | 65 (87.8) |  | 58 (86.6) | 62 (82.7) |  | 72 (93.5) | 68 (89.5) |
| Age (years) | **58.6 ± 13.9** | **62.8 ± 12.5^*^** |  | 60.3 ± 13.0 | 59.9 ± 14.4 |  | 54.3 ± 13.5 | 52.5 ± 14.6 |
| BMI (kg/m^2^) | 28.1 ± 5.1 | 27.4 ± 5.1 |  | **27.4 ± 5.9** | **26.0 ± 5.5^*^** |  | 27.1 ± 5.0 | 27.0 ± 4.4 |
| SBP (mmHg) | **146 ± 24** | **156 ± 24^**^** |  | 137 ± 27 | 140 ± 25 |  | 145 ± 23 | 147 ± 21 |
| DBP (mmHg) | 84 ± 13 | 85 ± 11 |  | 77 ± 16 | 79 ± 17 |  | 86 ± 11 | 85 ± 11 |
| Current smoking, n (%) | 11 (14.1) | 12 (16.2) |  | **10 (14.9)** | **23 (29.9)^*^** |  | 10 (13.0) | 9 (12.0) |
| Current alcohol consumption, n (%) | 26 (33.3) | 35 (46.6) |  | 11 (16.7) | 21 (27.3) |  | 33 (42.9) | 31 (41.3) |
| Number of comorbidities | 1 (1-2) | 1 (1-2) |  | 1 (1-2) | 1 (1-2) |  | 1 (1-2) | 1 (1-2) |
| Comorbidities, n (%) |  |  |  |  |  |  |  |  |
| - Hypertension | 63 (80.8) | 63 (85.1) |  | 44 (65.7) | 51 (66.2) |  | 60 (76.9) | 61 (80.3) |
| - Diabetes Mellitus | 21 (26.9) | 19 (25.7) |  | 18 (26.9) | 17 (22.1) |  | 14 (17.9) | 15 (19.7) |
| - History of coronary artery disease | 18 (23.1) | 15 (20.3) |  | 16 (23.9) | 17 (22.1) |  | **12 (15.4)** | **6 (7.9)^*^** |
| - Heart failure | 6 (7.7) | 6 (8.1) |  | 4 (6.0) | 6 (7.8) |  | **1 (1.3)** | **4 (5.3)^*^** |
| - Chronic lung disease | 9 (11.5) | 7 (9.5) |  | 6 (9.0) | 8 (10.4) |  | 2 (2.6) | 4 (5.3) |
| - History of malignancy^1^ | 11 (14.1) | 9 (12.2) |  | 14 (20.9) | 19 (24.7) |  | 10 (12.8) | 12 (15.8) |
| - Auto-immune disease | 2 (2.6) | 1 (1.4) |  | 2 (3.0) | 3 (3.9) |  | 6 (7.7) | 4 (5.3) |
| Lymphocytes (10^9^/L) | 1.6 (1.2-2.0) | 1.5 (1.2-2.0) |  | 1.2 (0.9-1.6) | 1.2 (0.8-1.7) |  | 1.5 (1.1-2.4) | 1.4 (1.3-1.8) |
| eGFR (ml/min/1.73m^2^) | 18.3 ± 5.7 | 17.1 ± 6.5 |  | - | - |  | 53.2 ± 18.5 | 51.8 ± 21.3 |
| Primary renal diagnosis, n (%) |  |  |  |  |  |  |  |  |
| - Primary glomerulonephritis | 8 (10.8) | 10 (16.7) |  | 7 (11.9) | 7 (10.8) |  | **14 (17.9)** | **21 (27.6)^*2^** |
| - Pyelonephritis | 1 (1.4) | 0 |  | 1 (1.7) | 0 |  | 0 | 2 (2.6) |
| - Interstitial nephritis | 5 (6.8) | 2 (3.3) |  | 2 (3.4) | 2 (3.1) |  | 5 (6.4) | 1 (1.3) |
| - Familial/hereditary renal diseases | 17 (23.0) | 8 (13.3) |  | 11 (18.6) | 8 (12.3) |  | 14 (17.9) | 17 (22.4) |
| - Congenital diseases | 4 (5.4) | 2 (3.3) |  | 3 (5.1) | 2 (3.1) |  | 5 (6.4) | 5 (6.6) |
| - Vascular diseases | 13 (17.6) | 18 (30.0) |  | 13 (22.0) | 14 (21.5) |  | 6 (7.7) | 7 (9.2) |
| - Secondary glomerular/systemic disease | 2 (2.7) | 2 (3.3) |  | 1 (1.7) | 6 (9.2) |  | 7 (9.0) | 1 (1.3) |
| - Diabetic Kidney Disease |  |  |  | 11 (18.6) | 10 (15.4) |  | 1 (1.3) | 3 (3.9) |
| - Other | 17 (23.0) | 12 (20.0) |  | 9 (15.3) | 14 (21.5) |  | 10 (12.8) | 10 (13.2) |
| - Unknown | 7 (9.0) | 15 (20.3) |  | 58 (86.6) | 63 (81.8) |  | 16 (20.5) | 9 (11.8) |
| Dialysis characteristics, n (%) |  |  |  |  |  |  |  |  |
| - Hemodialysis |  |  |  | 55 (82.1) | 54 (70.1) |  |  |  |
| - Peritoneal dialysis |  |  |  | **12 (17.9)** | **23 (29.9)^*^** |  |  |  |
| - Time on dialysis (months) |  |  |  | 33.5  (9.8-79.2) | 29.0  (16.0-68.5) |  |  |  |
| Transplant characteristics |  |  |  |  |  |  |  |  |
| - First kidney transplant, n (%) |  |  |  |  |  |  | 63 (80.8) | 61 (80.3) |
| - Time after last transplantation (years) |  |  |  |  |  |  | 7.0  (3.8-13.3) | 9.0  (4.0-19.0) |
| - Last transplant |  |  |  |  |  |  |  |  |
| - - Living, n (%) |  |  |  |  |  |  | 53 (67.9) | 55 (72.4) |
| - - Pre-emptive, n (%) |  |  |  |  |  |  | 28 (35.9) | 24 (31.6) |
| Number of immunosuppressive agents |  |  |  |  |  |  | **3 (2-3)** | **2 (2-3)^*^** |
| Immunosuppressive treatment at baseline, n (%) |  |  |  |  |  |  |  |  |
| - Steroids |  |  |  |  |  |  | **61 (78.2)** | **66 (86.8)^*^** |
| - Azathioprine |  |  |  |  |  |  | 16 (20.5) | 14 (18.4) |
| - Mycophenolate mofetil |  |  |  |  |  |  | **46 (59.0)** | **34 (44.7)^*^** |
| - Calcineurin inhibitor |  |  |  |  |  |  | 60 (76.9) | 61 (80.3) |
| - mTor inhibitor |  |  |  |  |  |  | **11 (14.1)** | **3 (3.9)^**^** |
| - Other |  |  |  |  |  |  | 1 (1.3) | 1 (1.3) |
| - Induction with rituximab last year, n (%) |  |  |  |  |  |  | 0 | 1 (1.3) |
| Received kidney transplant after baseline, n (%) | **4 (5.1)** | **9 (12.2)^*^** |  | **2 (3.0)** | **11 (14.3)^**^** |  | 0 | 1 (1.3) |
| Start dialysis after baseline, n(%) | **1 (1.3)** | **8 (10.8)^**^** |  | - | - |  | 0 | 0 |
| S1 specific antibody level at day 28 (BAU/mL) | **2106**  **(1027-4403)** | **2845^**^**  **(1713-4962)** |  | 1418  (628-2878) | 1752^*^  (935-3414) |  | 363  (93.1-827) | 281  (28.5-1218) |
| Variables are presented as mean ± SD, as median (IQ interval) in case of non-normal distribution, or otherwise stated*.*  *Abbreviations are:* CKD, chronic kidney disease; KTR, kidney transplant recipient; BMI, body mass index; SBP, systolic blood pressure; DBP, diastolic blood pressure; eGFR, estimated glomerular filtration rate.  ^1^ Including melanomas, excluding all other skin malignancies  ^2^ P=0.13 across all categories  ^*^ P<0.2  ^**^P<0.05 | | | | | | | | |

**Figure S1.** Association between S1-specific IgG antibody levels at 6 months and 28 days. Dotted lines indicate threshold for seropositivity (≥10 BAU/mL). Straight line indicates line of identity. Association was calculated using Pearson correlation.

**Figure S2.** Correlation between S1 specific IgG levels and neutralization against A) the ancestral SARS-CoV-2, B) Delta variant and C) Omicron variant of SARS-CoV-2, 28 days (upper panel) and 6 months (lower panel) after vaccination. Dotted horizontal line indicates the lower limit of detection (LLoD) of the level of neutralization (titer of 20) and dotted vertical line indicates threshold of seropositivity (≥10 BAU/mL). Association was calculated using Pearson correlation.

**Figure S3.** Association between S1 specific IgG level and IFNy levels A) 28 days and B) 6 monthsafter vaccination. Dotted horizontal line indicates the threshold for a detectable T-cell response (≥0.15 IU/mL) and dotted vertical line indicates threshold of seropositivity (≥10 BAU/mL).
